# The Present Discontent.—II

**Published:** 1906-10-27

**Authors:** 


### The Hospital

A Journal of

The Medical Sciences and Hospital Administration.

Vol. XLI.—No. 1,049. SATURDAY, OCTOBER 27, 1906.

### THE PRESENT DISCONTENT.—II.

In our last issue we made reference to certain expressions of dissatisfaction and uneasiness in regard to the present conditions of medical practice which have found a voice both in the medical and the lay Press. Allowing that there was some substantial ground for the existing discontent, we urged the great importance of seeking a remedy in the right quarter, and we gave reasons for believing that effective help was not to be obtained either from Parliament or the General Medical Council. With the latter, as we believe, there is little or no power to render the assistance that is desired, whilst the willingness of Parliament to fight the battles of the medical profession rests upon an assumption that is altogether devoid of foundation. There is, in short, no effective body of public opinion in favour of legislation to "put down" unqualified practice, even if it were possible to create one such an end could be accomplished by legislative methods. If the position is as we have defined it, ithere follows the inevitable conclusion that the profession must seek for a solution of its present difficulties, not from without, but from within. Instead of crying to Jupiter for assistance it must learn to put its shoulder to the wheel. Its ends can only be attained by a resolute determination on the part of the profession to act together and on lines which are determined by wide and large considerations arising from a study of what is practicable.

In this respect the elaborate and extensive organisation of the British Medical Association comes into view. The recent changes in its constitution have given it a distinctly democratic character, so that the most obscure member can find a platform on which he may initiate and advocate any desired reform. And still more, the voice of the profession in any district can, if it will express itself, find means to make itself heard even in the innermost councils of the organisation. Yet it is an open secret that the local or divisional meetings, as they are called, draw but scanty audiences, and that large numbers of the profession do not even belong to the Association. A similar apathy is manifested in regard to the medical defence associations and in the election of the direct representatives on the General Medical Council. For some reason or other the medical profession seems destitute of a genius for organisation, and no leader has yet arisen capable of uniting its scattered hosts. This is the more to be wondered at, seeing how special is the position of the profession in the body politic, and how great is its power in reference to some of its more serious hardships. Take for example the question of hospital abuse. It is a constant subject for professional complaint, and, as we pointed out on page 59 of our issue of last week, is very largely within professional control; yet it remains without remedy. We mentioned that, were the visiting staffs of the hospitals moved by the general practitioners to determine that the excess of hospital free medical relief must cease, the present abuses would disappear once for all.

In saying what we have done of the British Medical Association as the one organisation capable of collecting and expressing professional opinion, it must not by any means be concluded that we approve without qualification the policy of the Association. How far the official leaders of the Association may remove themselves from the general body of their *confrères* was made evident by the recent attempt to demand from candidates for election to the General Medical Council a pledge, the acceptance of which would have made the direct representatives mere delegates of the Association. To this at the time we offered decided opposition, and as the significance of the attempt became appreciated in the profession so strong a feeling was aroused that the proposal had to be abandoned. The lesson from this is not that the Association should be discarded, but that in its activities the general body of the profession should take a sustained and continuous interest. Our wish is to see the Association representative of the whole profession, for through it professional opinion and action may be strong because united; and we have full confidence that an organisation fully expressive of the medical profession will be guided by the highest and most worthy motives.

We will suggest one other direction in which the practitioner may seek help in his difficulties. Public appreciation and recognition rest, after all, on the ability to render public service. The claims of medical work in its more recent developments cannot be ignored, and with full appreciation of the difficulties of the situation we feel that an occasional course of post-graduation study is for many of us a necessity of the hour. The educative and stimulating influence of this is not to be despised, and we believe the day is not distant when its commercial value will also receive wide recognition. The more effective the practitioner's work, the more fully must he find both public confidence and material success.

